# Acute Colitis Induces Neurokinin 1 Receptor Internalization in the Rat Lumbosacral Spinal Cord

**DOI:** 10.1371/journal.pone.0059234

**Published:** 2013-03-21

**Authors:** Ming-Ming Zhang, Wei Ji, Li-Yu Pei, Wen Wang, Tao Chen, Wei Wang, Hui Li, Ting Zhang, Sheng-Xi Wu, Yun-Qing Li

**Affiliations:** 1 Department of Anatomy, Histology, Embryology & K. K. Leung Brain Research Centre, The Fourth Military Medical University, Xi’an, PR China; 2 Department of Orthopedics, Navy General Hospital of the People’s Liberation Army, Beijing, PR China; University of Cincinnatti, United States of America

## Abstract

Substance P (SP) and its receptor, the neurokinin 1 receptor (NK1R), play important roles in transmitting and regulating somatosensory nociceptive information. However, their roles in visceral nociceptive transmission and regulation remain to be elucidated. In the previous study, moderate SP immunoreactive (SP-ir) terminals and NK1R-ir neurons were observed in the dorsal commissural nucleus (DCN) of the lumbosacral spinal cord. Thus we hypothesized that the SP-NK1R system is involved in visceral pain transmission and control within the DCN. The acute visceral pain behaviors, the colon histological changes and the temporal and spatial changes of NK1R-ir structures and Fos expression in the neurons of the DCN were observed in rats following lower colon instillation with 5% formalin. The formalin instillation induced significant acute colitis as revealed by the histological changes in the colon. NK1R internalization in the DCN was obvious at 8 min. It reached a peak (75.3%) at 30 min, began to decrease at 90 min (58.1%) and finally reached the minimum (19.7%) at 3 h after instillation. Meanwhile, formalin instillation induced a biphasic visceral pain response as well as a strong expression of Fos protein in the nuclei of neurons in the DCN. Finally, intrathecal treatment with the NK1R antagonist L732138 attenuated the NK1R internalization, Fos expression and visceral nociceptive responses. The present results suggest that the visceral nociceptive information arising from inflamed pelvic organs, such as the lower colon, might be mediated by the NK1R-ir neurons in the DCN of the lumbosacral spinal cord.

## Introduction

Visceral pain occurs after mechanical or chemical stimulation in and around the internal organs. In contrast to somatic pain, visceral pain is difficult to localize and is often described as “deep pressure,” “cramping,” “spasms” or “squeezing”. The study of visceral pain is far behind that of somatic pain because it is difficult to access internal organs [1], [2] and the pathway of visceral noxious information transmission is complicated and remains largely unrevealed by current research techniques [3], [4].

Substance P (SP), a polypeptide consisting of 11 amino acids, is synthesized in approximately 20∼30% of the small or middle-size neurons in the dorsal root ganglia (DRG) [5]. The biological actions of SP are mediated via the neurokinin 1 receptor (NK1R), which belongs to the G-protein-coupled receptor (GPCR) family. Previous studies have shown that SP and NK1R are involved in the transmission of nociceptive information and the modulation of nociceptive pathways in the spinal cord [6], [7].

Morphological studies have revealed that SP-immunoreactive (SP-ir) fibers and terminals and NK1R-ir neurons are abundant in the spinal dorsal horn (SDH) [8]. Somatic noxious stimulation can induce robust SP release and the obvious internalization of NK1R into the neuronal cytoplasm within the superficial layers (laminae I–III) of the SDH [9]. As a common feature of GPCRs, internalization might serve as a reliable marker for the activation of NK1R-containing neurons [9], [10]. SP and NK1R are the main focuses of the current somatic pain studies, but their roles in visceral inflammatory pain, especially on pelvic organs, have not yet been revealed.

Our previous studies have indicated that the dorsal commissural nucleus (DCN), which is located dorsally to the central canal in the lower lumbar and sacral spinal cord segments, receives nociceptive information from the pelvic organs and plays an important role in visceral nociceptive transmission and regulation [11]. It has also been confirmed that moderate SP-ir fibers and NK1R-ir neurons are distributed in the DCN [12].

The present study is thus designed to investigate the involvement of the SP-NK1R system in pelvic visceral noxious transmission and modulation. The noxious behavioral responses, histological changes in the lower colon and the temporal and spatial features of NK1R internalization and Fos expression in the DCN were observed following formalin instillation into the rat lower colon.

## Materials and Methods

### Animals

Adult male Sprague Dawley rats weighing 220–250 g were used. The animals were acclimated to the laboratory environment for 5–7 d before use. While in their home cage environment, they were allowed free access to a standard rat diet and tap water. The room was maintained at 20–23°C with a 12 h/12 h light/dark cycle. The experimental procedures were approved by the Animal Care and Use Committee of the Fourth Military Medical University (Xi’an, P.R. China). All effort was made to minimize both the number of animals used and their suffering. The rats were anesthetized with intraperitoneal (i.p.) injection of sodium pentobarbital for all of the surgical procedures except for the formalin instillation.

### Formalin Instillation

The stimulus used in the experiments was instillation of the colon with dilute formalin or isotonic saline in control animals. Briefly, the animal was anesthetized with a small amount of halothane (induction at 3%, then 1.5% in a mixture of 2∶3 nitrous oxide and 1∶3 oxygen), which allowed for a prompt return to consciousness. We wrapped a surgical tape on a polyethylene (PE) tube, about 30 mm from the edge. While under anesthesia, the rat was suspended by its tail (less than 5 min) and above specially designed PE tube, which insured that the tube inserted to the same position and avoided solution overflowing, was inserted through the anus. Then, the formalin instillation was performed as slowly as possible through the tube. It consisted of 100 µl of either 5% formalin solution or saline (0.9% NaCl) both supplemented with 1% Evans blue (w/v) to verify at the end of the test that no luminal leakage had occurred at the time of instillation and that the instillation had not been transmural.

### Behavioral Tests

The rats were randomly divided into 6 treatment groups and marked with “For” “Sal” “L+F”“F+L” “S+F” or “Nai”. The “L+F”, “F+L” and “S+F” groups received intrathecal catheterization 5 d prior to the formalin instillation. All animals were then placed into observation boxes for 30 min to adapt to the surrounding environment prior to the behavioral experiments. The PE tube was connected with a 50 µl volume microinjector. For the “L+F” and “F+L” groups, 10 microliters of vehicle (70% DMSO; Sal) containing L732138 (100 nmol) were slowly injected, then the tubes were flushed with 5 µl saline. The injection process was finished within one min. The “S+F” group was given an equal dose of saline as intrathecal vehicle treatment control group. The “For” group was given 100 µl of 5% formalin into the colon, and the “Sal” group was given an equal dose of saline as negative control. The “L+F” group was pretreated with L732138 (100 nmol/10 µl) 10 min prior to formalin administration, the “S+F” group was pretreated with saline 10 min prior to formalin administration and the “F+L” group was post-treated with L732138 60 min after formalin administration; The “Nai” group was used as a negative control with no drug applied. After recovering consciousness, the rats were observed for 3 h to record visceral pain-related behaviors.

The visceral pain behavior score was analyzed according to the method described by Miampamba [13], including abdominal licking (L), backward extension (B), contraction of the flanks (C) and whole body contraction (W). The whole body contraction was further divided into three categories depending on the length of the contraction: shorter than 30 sec (W1), 30–60 sec (W2) or longer than 1 min (W3). The visceral pain score (PS) was calculated every 15 min during the 180 min observation window according to the following formula [13]:
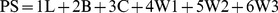



### Intrathecal Catheterization

Under pentobarbital anesthesia, a dorsal midline incision (3 cm) was made at the level of thoracic vertebrae 3–4. A PE tube was introduced into the subarachnoid space of the lumbar enlargement and extended to lumbar vertebrae 1–2. The tube was then brought out of the neck skin and secured *in situ*, filled with artificial cerebrospinal Fluid (ACSF: 124 NaCl, 2.5 KCl, 2 CaCl_2_, 2 MgSO_4_, 25 NaHCO_3_, 1 NaH_2_PO_4_, and 10 glucose, pH 7.4, in mM) and sealed with heat. The rats were allowed to recover for a period of 3–5 d before further investigation. Only the animals judged to be neurologically normal were used for the following experiments. At the end of the experiments, the animals were dissected to confirm the position of the intrathecal catheterization and that there was no spinal injury or remote hemorrhage.

### Haematoxylin and Eosin (HE) Staining

For histological examination, lower colon tissues were taken from an area with apparent inflammation and randomly from areas in other treatment groups and placed in cold 4% paraformaldehyde in a 0.1 M phosphate buffer (PB, pH 7.4) overnight. After 48–72 h, the tissues were embedded in paraffin, cut on a microtome (Kryostat 1720; Leitz, Mannheim, Germany) and stained conventionally with HE.

### NK1R- and Fos-immunostaining

The rats were divided into 6 groups as described above. After recovering consciousness, the rats were placed in the observing boxes for another 3-h observation.

At 8 min, 10 min, 15 min, 30 min, 90 min and 120 min after formalin instillation, the rats were deeply re-anesthetized with an overdose of sodium pentobarbital (100 mg/kg, i.p.) and perfused with 200 ml of 0.9% saline, followed by 500 ml of 0.1 M PB containing 4% (w/v) paraformaldehyde. After perfusion, the spinal cords were removed immediately and placed into 0.1 M PB containing 30% (w/v) sucrose overnight at 4°C, and cut into 20 µm-thick serial frontal sections on a freezing microtome (Kryostat 1720; Leitz, Mannheim, Germany). The sections were collected in sequence, divided into 2 series and then washed with 0.01 M phosphate buffer solution (PBS, pH 7.4). The first series of sections were incubated respectively with rabbit anti-NK1R antiserum (1∶1000; Chemicon, Temecula, CA) and mouse anti-Fos antiserum (1∶500; Sigma, MO, USA) in PBS containing 5% (v/v) normal donkey serum (NDS), 0.3% (v/v) Triton X-100, 0.05% (w/v) NaN_3_ and 0.25% (w/v) carrageenan (PBS-NDS, pH 7.4) overnight at 4°C.

After washing with 0.01 M PBS, for NK1R immunofluorescence, the sections were incubated with Alexa 594-conjugated donkey anti-mouse IgG (1∶500; Sigma) secondary antibodies in PBS containing 5% normal goat serum and 0.2% Triton X-100 for 2 h. The sections were washed with 0.01 M PBS 3 times for 10 min each between each step. Finally, the slides were mounted onto gelatin-coated glass slides, air-dried and cover-slipped with a mixture of 50% (v/v) glycerin and 2.5% (w/v) triethylene diamine (anti-fading agent) in 0.01 M PBS. Images were obtained using a confocal laser scanning microscope (FV1000; Olympus, Tokyo, Japan).

For Fos immunohistochemistry, the sections were washed before being incubated with biotinylated anti-mouse IgG (1∶200, Sigma) for 2 h at room temperature (about 21°C). Sections were washed (3×10 min) in PBS before being incubated in avidin-biotin complex (ABC solution, Vector) for 30 min, then washed (3×10 min) again and reacted with 0.05 M of Tris–HCl buffer (pH 7.6) containing 0.04% diaminobenzidinetetrahydrochloride (DAB) (Dojin, Kumamoto, Japan) and 0.003% H_2_O_2_ for visualizing Fos (Fos-positive nuclei appeared black judged by eye). Sections were washed (once immediately after removing the DAB solution, then 3×10 min), then mounted on gelatin-coated glass slides and allowed to air-dry overnight. Sections were dehydrated in increasing concentrations of ethanol, cleared, and coverslipped for light microscopic visual examination (AHBT3; Olympus, Tokyo, Japan).

The second series of sections were incubated respectively with normal rabbit serum or mouse serum instead of the primary antibody followed by the same staining protocols to serve as negative controls. The intensity of the staining was quantified using densitometry by experimenters who were blind to the treatment conditions.

### Quantification of Fos Expression

The counts of Fos immunoreactive (Fos-ir) neurons in the spinal cord were represented as the average number of neurons per section. We restricted the analysis to the L6 segment because it had the largest numbers of immunolabeled neurons in response to the acute colitis-induced pain. We counted the Fos-ir cells in the intermediolateral nucleus (IML), SDH and DCN. The number of labeled neurons was estimated from the counts of positively stained cells in a minimum of 6 sections per spinal cord segment. To avoid counting a neuron more than once, the sections used for the counts were separated by at least 100 µm. The differences in cell counts were evaluated statistically using an analysis of variance (multivariate with repeated measures) to examine the interactions among each group. The person counting cells was blind to the treatment condition.

### Quantification of NK1R Internalization

Receptor internalization was quantified in two ways: (1) the number of endosomes was counted, and neurons were scored as having internalization if more than 10 fluorescent endosomes were present in the cytoplasm in a single optical section (0.5-µm thick); (2) The proportion of NK1R fluorescence in the cytoplasm was quantified in immunoreactive neurons chosen at random. For this quantification, a single confocal image that included the nucleus and a large area of cytoplasm was selected, and the image was analyzed using Adobe Photoshop 7.0 (Adobe Systems Inc., Mountain View, Calif., USA). Because the nucleus does not contain NK1R, the maximum pixel value (black = 0, white = 255) was determined for the nucleus. This represented the level of background fluorescence, and this value was used as the threshold when determining the degree of immunoreactivity for the neuron. A line was drawn around the outside of the cell, and the total cell fluorescence (surface plus cytoplasm) was measured as the number of pixels with intensities above the threshold. To measure the number of surface and cytoplasmic receptors separately, a second line was drawn inside the cell membrane 0.5 µm in from the first line, and the number of pixels with intensities above the threshold in the cytoplasm only was determined. The percentage of fluorescence present in the cytoplasm was then calculated as the second measure (immunoreactivity in the cytoplasm only) divided by the first measure (total immunoreactivity).

The amount of NK1R internalization was quantified using a standard method. This consisted of visually counting the number of NK1R-ir neurons in the DCN, with and without NK1R internalization, to calculate the percentage of NK1R-ir neurons in which internalization occurred. The neuronal somas and contiguous proximal dendrites with ≥10 endosomes with diameters of 0.1–0.7 µm were considered to have internalized receptors [7]. The person counting the neurons was blind to the treatment condition.

### Statistical Analysis

All data were collected by experimenters who were blind to the surgical and chemical treatments. Statistical analysis was performed using SPSS software (version 13). Data were expressed as the mean ± standard error of the mean (mean ± SEM). A one-way analysis of variance (ANOVA) followed by post hoc Fisher’s LSD Test was used for data analysis.

## Results

### Formalin-induced visceral Pain Behaviors

**Figure 1:**
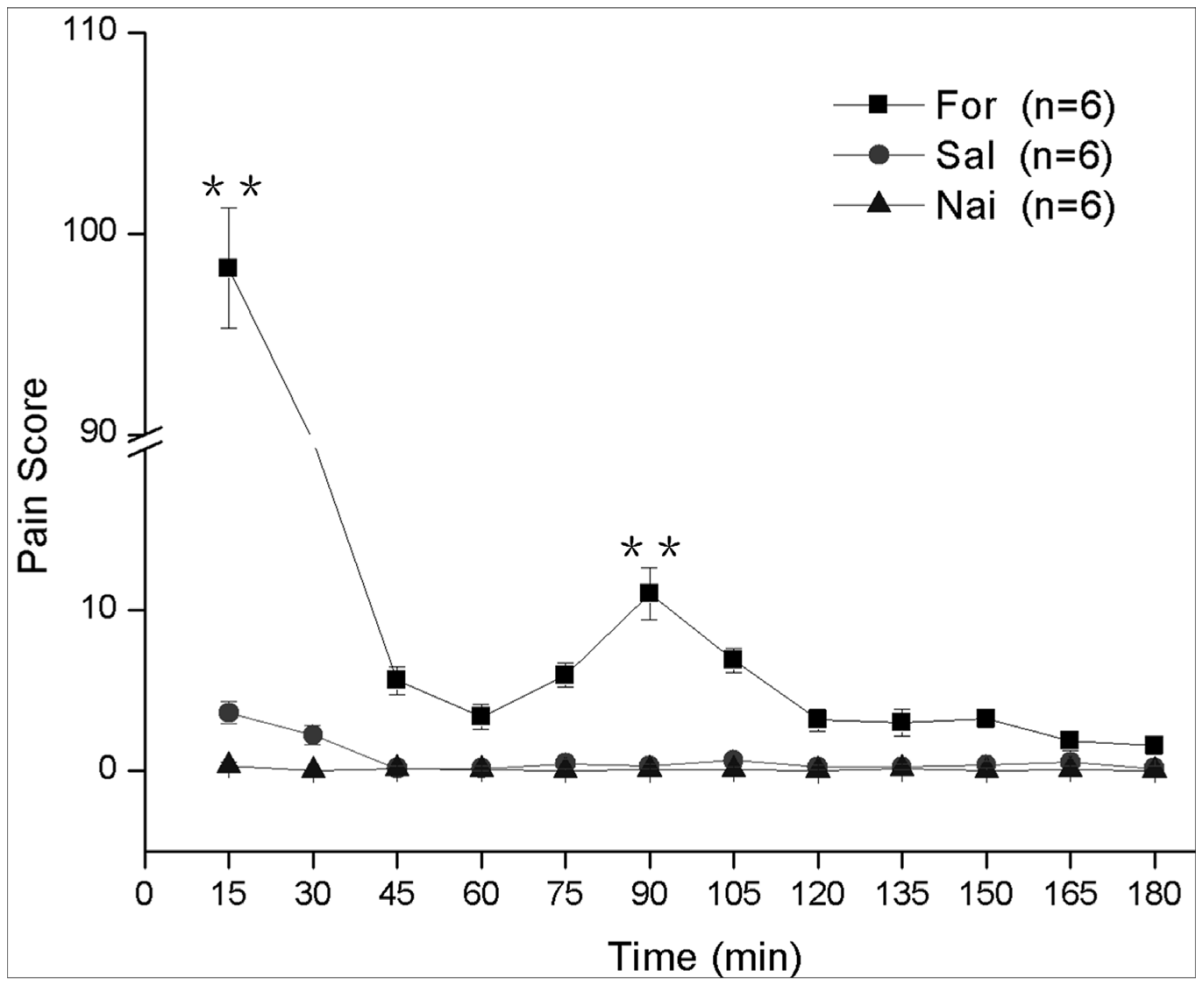
The line graph shows the time course of pain scores in responses to formalin instillation (“For”, ▪), saline instillation (“Sal”, •) and naïve group (“Nai”, ▴). ** *P*<0.001 formalin instillation *vs.* saline instillation or naive group. n = 6 in each group. Values are means ± S.E.M.

After formalin instillation, animals showed a two-phase visceral pain response. The first phase reached a peak within 30 min following injection (maximum PS = 98±6) that was approximately 24 times larger than that of the saline instillation or naive group (*P*<0.001 formalin instillation *vs.* saline instillation or naive group). A second PS peak occurred 90 min after injection (PS = 10±2) and was about five times larger than that of the saline instillation or naive group (*P*<0.001 formalin instillation *vs.* saline instillation or naive group) (Figure 1). The total number of visceral pain behaviors [13], [14], [15] also exhibited biphasic pattern of pain sensitivity which occurred within the first 30 min (*P*<0.001 formalin instillation *vs.* saline instillation or naive group) and 90 min (*P*<0.001 formalin instillation *vs.* saline instillation or naive group) (File S1; Figure S1).

### Formalin-induced Colitis: HE Staining

**Figure 2:**
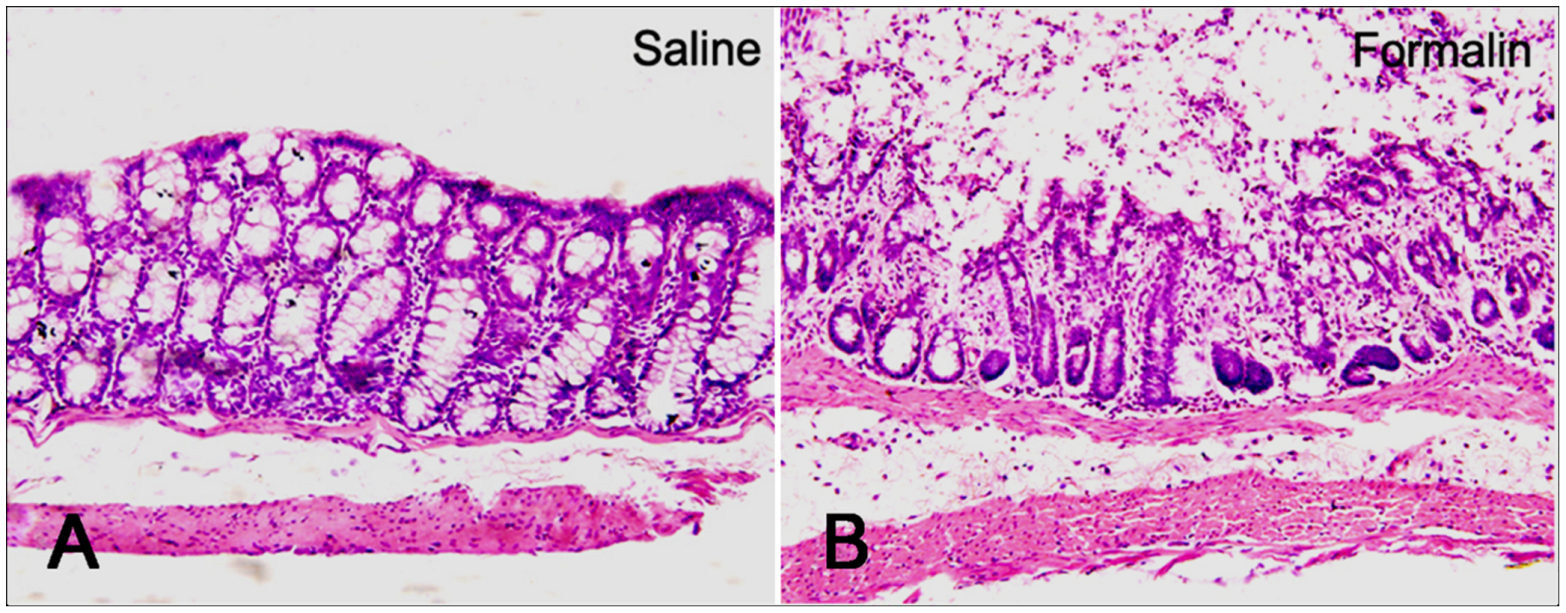
Histological appearance of the colonic wall in rats subjected to saline (A) or formalin (B). There is no significant histological modification was observed in the tissue exposed to saline (**A**). After formalin instillation, the inflamed area showed ulceration covered by exudates with polymorphonuclear cells. Necrosis restricted to the mucosal layer and the loss of crypt epithelial cells can be observed. A mild cellular infiltration in the lamina propria was also noted, while the muscularis mucosae were normal. These features were associated with marked submucosal edema and dilated capillaries (**B**).

Either 5% formalin (colitis model) or normal saline (control) were instilled into the lower colon through above mentioned PE tube. Three hours later, the lower colon tissues were stained with HE. The saline instillation group showed no morphological tissue damage when observed under a light microscope (Figure 2A). Whereas in the formalin instillation group, the observed cellular infiltrate was most intense in the epithelial regions. Smooth muscle damage was also noticeable in the submucosa, while the mucosa and the muscularis externa did not exhibit any overt injury (Figure 2B).

### Formalin Instillation in the Colon Induced Fos Expression in the Lumbosacral Cord

**Figure 3:**
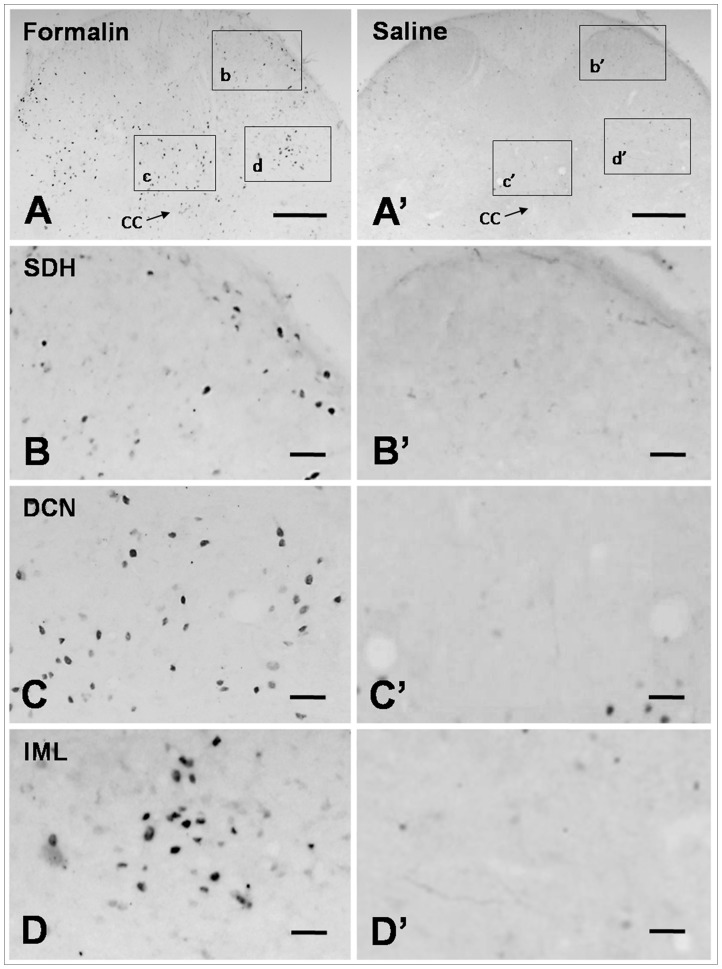
The results of Fos immunohistochemical staining. At 90 min after formalin instillation, the expression of Fos immunoreactive neurons was up-regulated in the spinal cord, and these neurons were mainly distributed in the DCN. Scale bars: 300 µm (**A, A’**); 50 µm (**B–D, B’–D’**).

**Figure 4:**
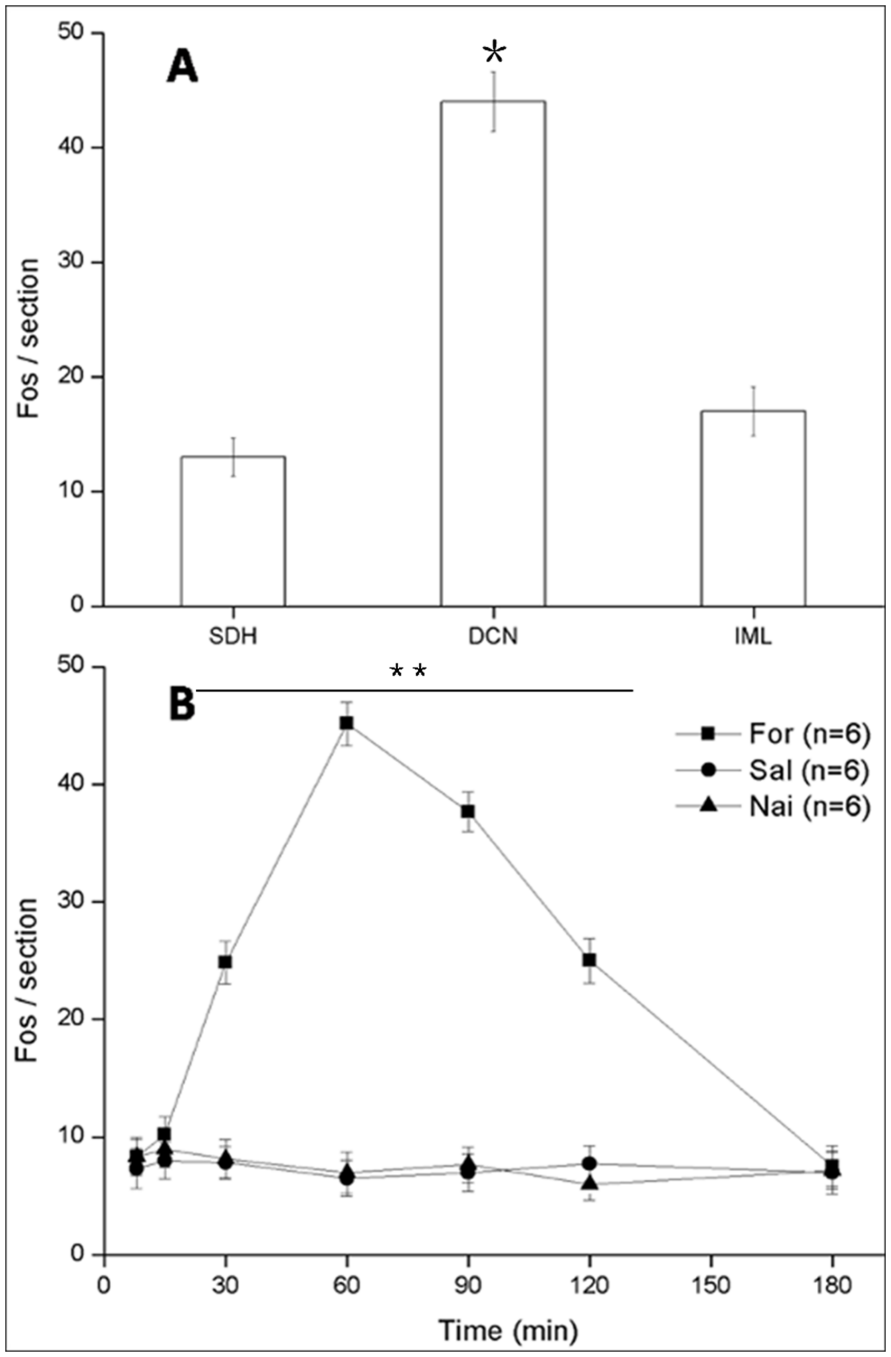
The Fos immunoreactive (Fos-ir) neurons mainly distributed in DCN in the spinal cord (A). *P<0.01 DCN *vs.* SDH or IML. The number of Fos-ir DCN neurons showed that in the formalin instillation group (“For”, ▪), Fos begin to increase 15 min after irritation and peaked at 60 min, then returned to normal compared to the saline instillation group (“Sal”, •) and naïve group (“Nai”, ▴) 3 h later (**B**). ** *P*<0.001 formalin instillation *vs.* saline instillation or naive group. n = 6 in each group. Values are means ± S.E.M.

The Fos protein is a widely used molecular marker that indicates the activation of neurons after somatic or visceral nociceptive stimuli [16]. We thus used Fos staining to determine whether DCN neurons were involved in formalin-induced colitis. The results of the present study showed that Fos was expressed in many neurons in the L6 and S1 spinal cord after formalin instillation. These Fos immunoreactive (Fos-ir) neurons were primarily distributed in the DCN, with a few cells also observed in the IML and the medial aspects of laminae I and II of SDH (P<0. 01 DCN *vs.* SDH or IML; Figures 3, 4A, S3). In the present study, we focused on visceral sensations in the lower colon where primary information mainly transmitted through pelvic nerve [17], [18]. Our results indicated that the DCN in lumbosacral might have closer relationship with pelvic visceral nociceptive transmission and modulation compared with SDH and IML. Our supplementary results also showed the weak expression of Fos and NK1R in SDH at the level of thoracolumbar under the condition of acute colitis induced by formalin instillation (File S1; Figure S3). These results supported the important role of DCN in acute pelvic visceral pain. Thus, the immunological results were analyzed in the DCN at the level of lumbosacral only. Previous study indicated that transcriptional activation of the gene c-fos occurs within minutes of stimulation, with the accumulation of mRNA reaching its peak approximately 30 to 40 min later [16]. In present research, Fos protein expression displayed a postpone compared with behavioral test. The number of Fos-ir neurons increased significantly at 15 min and reached the maximum at 60 min (44±4, per section). The number then decreased until 180 min (8±2 per section) (Figure 4B).

### Colon Formalin Instillation Induces NK1R Internalization in the DCN

**Figure 5:**
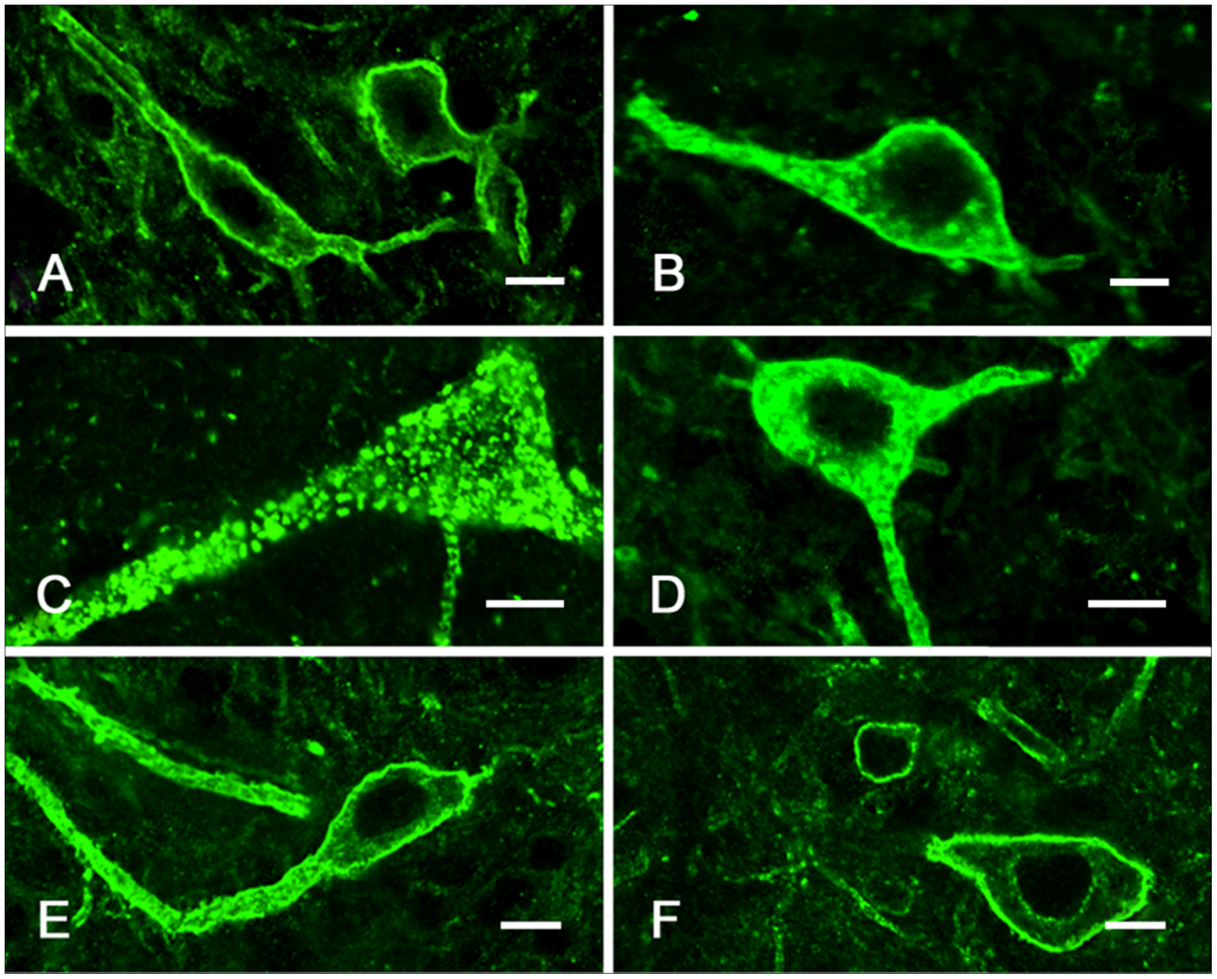
Confocal images of NK1R immunoreactivity in DCN. In the saline controls, NK1R was associated with the cell surface (**A**). Fifteen minutes after formalin stimulation, NK1R was present in the endosomes and mainly distributed in the dendrites (**B**). Thirty minutes after formalin instillation, the NK1R endosomes were mainly distributed throughout the cytoplasm (**C**). Sixty minutes after formalin stimulation, the NK1R endosomes were primarily located in the dendrites rather than the cell body (**D**). Ninety minutes after formalin instillation, the NK1R endosomes were mainly restricted to the dendrites (**E**). After 3 h, most of the NK1R-ir was uniformly distributed at the plasma membrane (**F**). Scale bars, 10 µm.

We hypothesized that NK1R-ir neurons in the DCN might play a role in the regulation of formalin-induced inflammatory pain. In the control group, NK1R was predominantly distributed along the membrane of cell body and the initial segment of the dendrites [19] and endosomes were occasionally observed in the cytoplasm (less than 10) (Figure 5A). After formalin instillation, NK1R endocytosis was greatly enhanced in the DCN neurons, with clumped endosomes distributed in the cytoplasm and dendrites.

**Figure 6:**
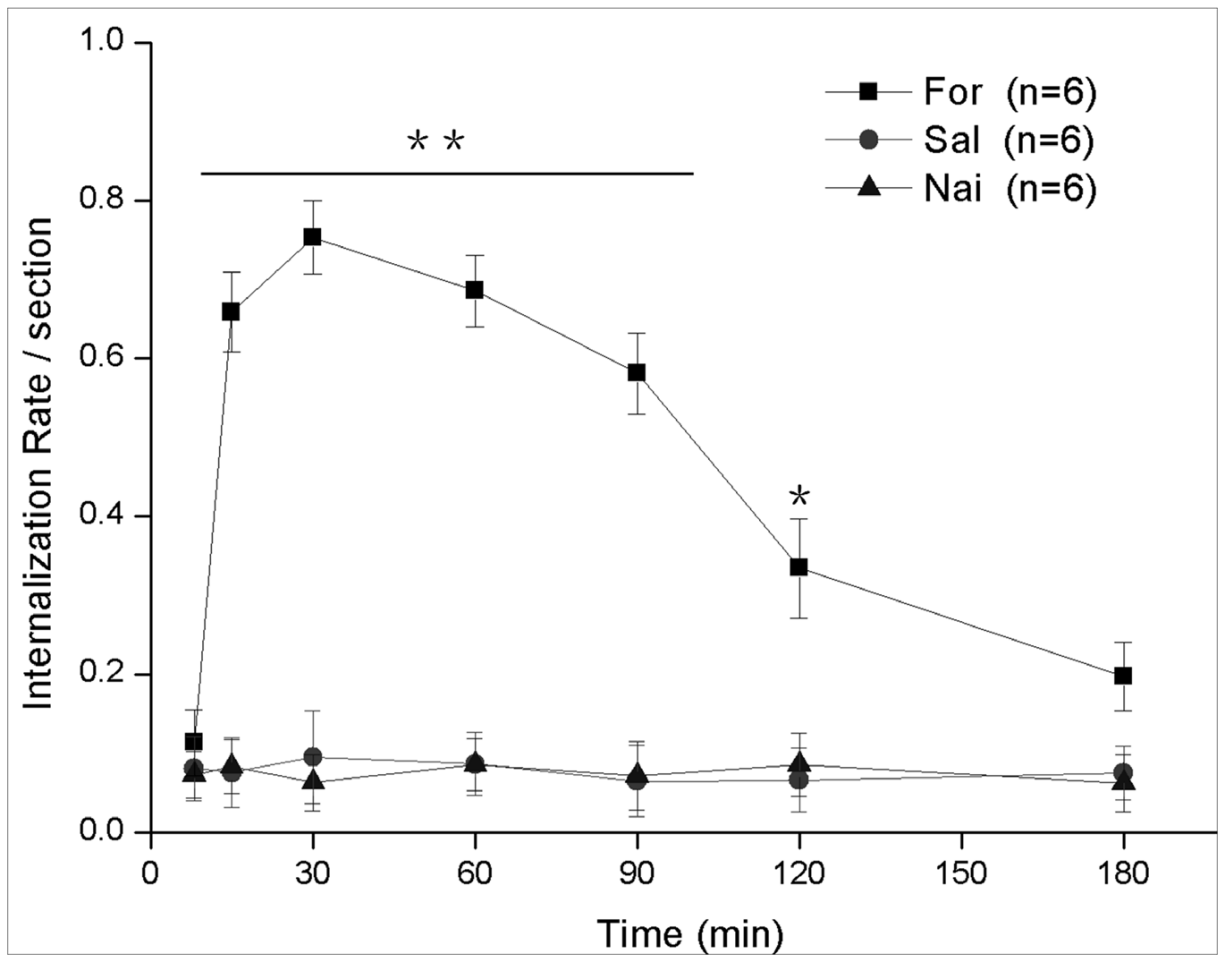
The temporal quantification of NK1R internalization in the DCN for the formalin instillation group (“For”, ▪), saline instillation group (“Sal”, •) and naïve group (“Nai”, ▴). * *P*<0.01 formalin instillation *vs.* saline instillation or naive group; ** *P*<0.001 formalin instillation *vs.* saline instillation or naive group. *n* = 6 in each group. Values are means ± S.E.M.

Eight minutes after formalin stimulation, NK1R internalization was observed in 16.3±2.6% NK1R-ir neurons in the DCN of the lumbosacral cord. The endosomes were primarily distributed within the dendrites, with a few in the cell body (Figure 6). At 15 min, internalization occurred in approximately 65.8±4.7% of NK1R-ir neurons, in which the endosomes were mainly distributed in the dendrites with discontinuity in the membranes and homogenously in the cytoplasm (Figures 5B, 6). At 30 min, internalization reached the peak, with 75.3±7.2% NK1R-ir neurons showing obvious internalization in which the endosomes were distributed throughout the cytoplasm and in the cell body (*P*<0.001 formalin instillation *vs.* saline instillation or naive group, Figures 5C, 6). At 60 min, some of the internalized NK1R were recycled to the membranes of the cell body, and the endosomes were primarily found in the dendrites rather than the cell body compared with the 30 min time point (Figures 5D, 6). At 90 min, the endosomes were mainly restricted to the dendrites and began to decrease in number, with 58.1±5.1% neurons showing internalization (Figures 5E, 6). Three hours after formalin stimulation, internalization was only observed in 19.7±5.3% neurons (Figures 5F, 6).

### The Intrathecal Injection of the NK1R Antagonist L732138 Affects Pain Behavior, NK1R Internalization and Fos Expression

**Figure 7:**
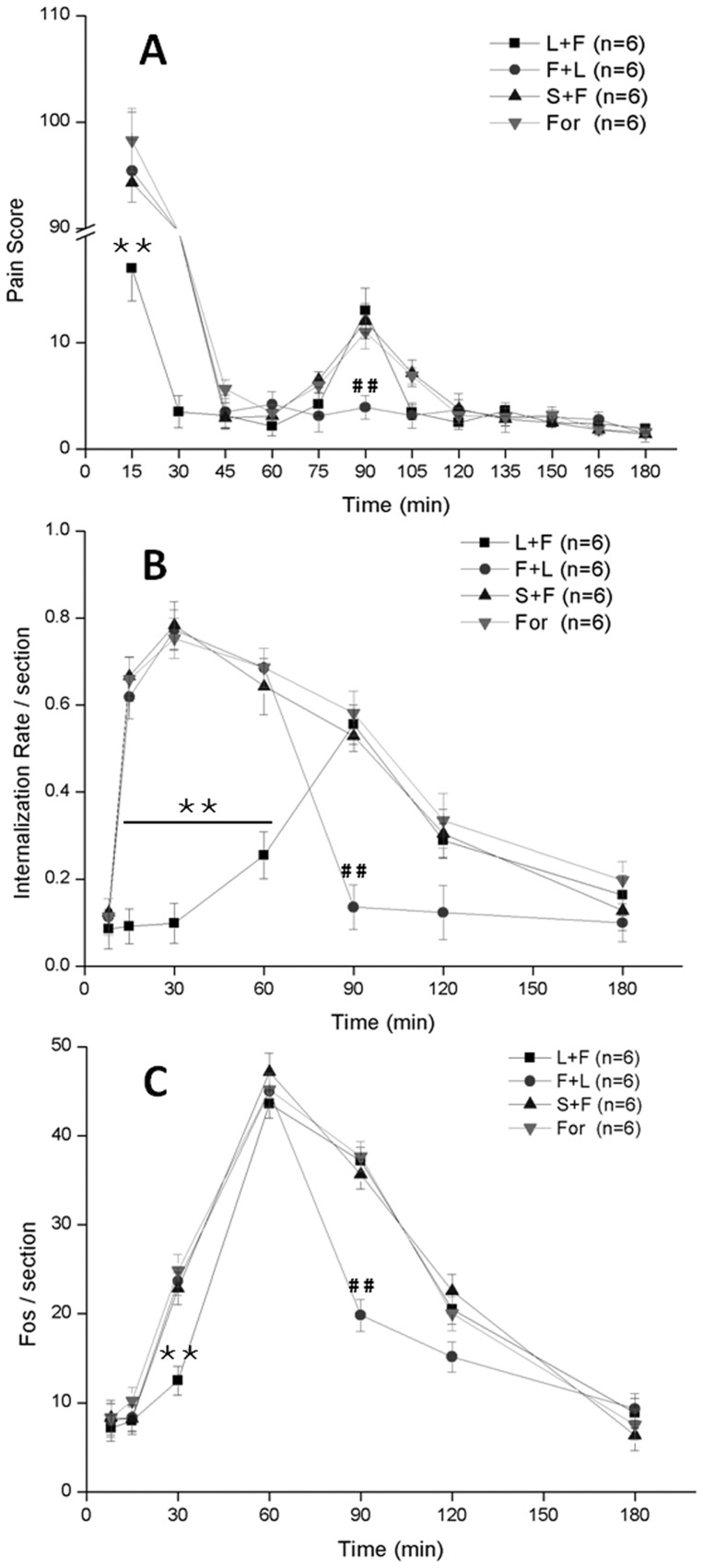
The effects of intrathecal L732138 injection on pain behavior (A), NK1R internalization (B) and Fos expression (C). ** *P*<0.001 L732138 pretreatment *vs.* saline pretreatment or L732138 post treatment or formalin instillation group; ^##^ *P*<0.001 L732138 post treatment *vs.* saline pretreatment or L732138 pretreatment or formalin instillation group. n = 6 in each group. Values are means ± S.E.M.

The NK1R antagonist L732138, an L-tryptophan derivative, is a competitive and selective antagonist for NK1R and attenuates hyperalgesia [20]. The intrathecal injection of L732138 (100 nmol/10 µl) 10 min prior to formalin instillation (L+F) significantly inhibited the first phase pain behavior, but had no effect on the secondary phase. The PS of the L732138 pretreatment group (L+F) decreased the first phase (18±3) to a level that was about five times smaller than that of saline pretreatment (S+F), L732138 post treatment (F+L) and formalin instillation group without pretreatment (For) (*P*<0.001 L732138 pretreatment *vs.* saline pretreatment or L732138 post treatment or formalin instillation group). We also found that the intrathecal injection of L732138 (100 nmol/10 µl) 60 min after formalin instillation (F+L) significantly reduced the second phase pain behavior. The PS of the L732138 injection group decreased the second phase (3±1) to a level that was about three times smaller than that of saline pretreatment (S+F), L732138 pretreatment (L+F) and formalin instillation group without the antagonist injection (For) (*P*<0.001 L732138 post treatment *vs.* saline pretreatment or L732138 pretreatment or formalin instillation group) (Figures 7A, S2). Our results suggested that intrathecal pre/post treatment with the NK1R antagonist L732138 attenuated nociceptive responses significantly.

It was also observed that L732138 regulated the NK1R internalization effect. Our results show that the intrathecal injection of L732138 10 min before formalin instillation significantly reduced the NK1R internalization in first 60 min from 75.3±7.2% to 21.1±4.6%. However, L732138 pretreatment had no effect on the receptor internalization during the following 2 h, while the L732138 injection 60 min after formalin instillation reduced NK1R internalization from the maximum 58.1±5.1% to 13.5±5.2% in following period (*P*<0.001 L732138 post treatment *vs.* saline pretreatment or L732138 pretreatment or formalin instillation group) (Figure 7B).

We then tested whether NK1R is involved in the regulation of Fos expression. We found that pretreatment with L732138 prior to formalin instillation can significantly reduce the number of Fos-ir neurons at 30 min after stimuli to approximately 50% less than the formalin instillation group (13±3, per section; *P*<0.001 L732138 pretreatment *vs.* saline pretreatment or L732138 post treatment or formalin instillation group). In addition, L732138 post-treatment reduced Fos expression significantly 90 min after formalin to 50% less than the formalin instillation group in following period (19±2, per section; *P*<0.001 L732138 post treatment *vs.* saline pretreatment or L732138 pretreatment or formalin instillation group) (Figure 7C).

## Discussion

In the present study, we used an acute colitis model to systemically analyze the spatial and temporal changes of NK1R and Fos in DCN neurons, which made detailed positioning of pelvic visceral (lower colon, rectum and bladder et al.) primary sensitive neurons in spinal cord based on previous study [21], [22]. Our major findings are: (1) colon formalin instillation induces significant NK1R internalization in the DCN; (2) formalin instillation causes strong Fos expression in the DCN region and a biphasic visceral pain behavioral response; (3) intrathecal pretreatment with the NK1R antagonist L732138 attenuates NK1R internalization, Fos expression and the nociceptive responses.

Instilling formalin into the rat lower colon can induce pain responses and could well mimic clinical pelvic visceral inflammatory pain [13]. In the present study, lower colon formalin instillation induced a two-phase visceral pain response that consisted of an acute phase (within the first 30 min) followed by a tonic phase (75–105 min), which showed a similar characteristic of formalin induced somatic pain except the prolonged time course [23]. The underlying mechanism of this delayed biphasic pain is unknown and worthy of extensive research. The morphological results showed that the NK1R internalization occurred began with onset of pain behavior in acute phase (30 min), with 75.3% of the NK1R-ir neurons contained endosomes, which were concentrated in cytoplasm, and persisted for an extended period of time with 58.1% of neurons contained endosomes which most restricted to dendrites. These findings highly suggest that NK1R internalization, which drives reversible structural changes in central nervous system neurons *in vivo* [24], is closely related to formalin-induced pelvic visceral pain behavior.

Previous studies of formalin-induced inflammation have demonstrated that the underlying mechanism of the first phase pain is the direct activation of nociceptive C fibers of which approximately 50% express SP [25], [26]. Noxious stimuli, including formalin instillation, could significantly induce SP release and activate postsynaptic NK1Rs, leading to NK1R internalization. SP release correlates with the intensity of noxious stimuli [27]. Recent studies have suggested that increasing the intensity of noxious stimuli causes C-fibers to fire at a high frequency or in bursts, which increases SP release and minimizes its degradation by peptidases, leading to a greater activation of NK1Rs [28]. Other reports have also indicated that NK1R internalization can only increase with SP concentration to the point where 100% of the NK1R neurons have internalized receptors, whereas the amount of released SP can increase well beyond this point [29], [30]. In the first phase of formalin-induced colitis pain, a robust stimulation could induce a quick and strong activation of NK1R internalization, as was observed in the present study. We also observed that the intrathecal injection of the NK1R antagonist L732138 10 min before noxious stimulation could successfully reduce the first phase of the pain response and NK1R internalization. These results further support our hypothesis that SP release and NK1R internalization play essential roles in formalin-induced pain behavior.

In addition to the rapid NK1R internalization, formalin instillation also induces a chronic and delayed NK1R internalization. From 75–105 min after formalin stimuli, endosomes are mainly restricted to the dendrites, and recycled NK1Rs are distributed in the membranes of the cell body. However, the mechanism of NK1R internalization in following period is undetermined. A previous study suggested that internalized NK1Rs, by forming a scaffolding complex with β-arrestin, activate an additional signaling pathway involving the tyrosine kinases, extracellular signal regulated kinase 1 and 2 (ERK1/2) and Src family kinase (SFK) [30]. A study in dorsal horn neurons indicated that NK1Rs internalized in the soma may send a signal to the nucleus to modulate gene expression, whereas NK1R internalized in the dendrites may produce other signals, such as the activation of NMDA receptors by Src [7]. The tyrosine phosphorylation of these NMDA receptors is regulated by the opposing actions of the SFK and protein tyrosine phosphatases (PTPs) and may be required to induce further SP release [31]. In the current study, the endosomes were mainly distributed in dendrites, rather than the soma, during the following period (90 min) after formalin instillation. Together with the above reports, this suggests that NK1Rs internalized at the beginning may activate an intracellular signaling pathway and modulated gene expression. Then, some of the internalized NK1R could be transmitted to dendrites to receive persistent nociceptive afferents and interact with NMDA receptors. It has been reported that NMDA receptors are expressed on SP-containing primary afferent terminals [32]. Using an NK1R antagonist or capsaicin to destroy SP-ergic fibers can block NK1R internalization in the spinal dorsal horn when NMDA is injected into the cerebral ventricle [33], [34]. Through the activation of NK1Rs, SP potentiates currents through NMDA receptors [35] and the NMDA-evoked cytoplasmic Ca^2+^ ([Ca^2+^]_cyt_) transient [36]. These data also suggest that C fibers remain activated during the secondary phase of visceral pain and may continually release SP in the DCN.

In addition to previous study that ablation of lamina I NK1R-ir spinal neurons could inhibit mechanical and thermal hyperalgesia [37], the intrathecal injection of the NK1R antagonist L732138 10 min prior to and 60 min after lower colon noxious stimulation was able to reduce the first and second phase of the pain response, respectively. Morphological staining showed that the Fos up-regulation is reduced by the application of the NK1R antagonist. Fos is a well-established marker of neural activity that transmits extracellular signals to intracellular cascades that modulate function [38], [39]. Our results are consistent with previous studies, in which NK1R antagonists reduced Fos expression after dorsal root injury and subcutaneous formalin injection (noxious stimuli) [40], [41], [42]. However, L732138 does not block the up-regulation of Fos in the DCN completely. These results could be explained by our morphological observation that NK1R is not completely co localized with Fos. It is consistent with previous report that after visceral stimuli, many Fos-immunopositive neurons in the spinal dorsal horn were immunoreactive for NK1R and a few were somatostatin (ss2A) positive. In contrast, with cutaneous stimulation, only NK1R-positive neurons showed Fos expression [43]. It was proposed that two separate hyperalgesia pathways exist, one of which is NK1 receptor dependent, whereas the other does not require intact substance P/NK1 signaling [44]. These results suggest that neurons which respond to visceral stimuli may receive other types of inputs from peripheral, local or supraspinal origins, such as somatostatinergic inputs. In addition, we found that Fos protein displayed a postpone compared with behavioral test. The underlying mechanism of this discrepancy should be investigated in our future research.

It is known that the administration of L-732138 exerts an attenuation of hyperalgesia [20] and it has been also described that L-732,138 is able to antagonize H(3) antagonist-induced skin vascular permeability [45]. In addition, antitumor activity of L732138 was shown [46].In present study, the injection of L732138 not only caused a significant decrease in NK1R internalization but also remarkably reduced visceral pain behavior and Fos expression. Thus NK1R antagonists may have effects on both pain behavior and Fos expression. Our results suggest the possibility of interfere with NK1R internalization in visceral pain research as well.

In conclusion, our study suggests that in the DCN of the spinal cord, SP and NK1R can mediate pelvic visceral inflammatory pain induced by formalin instillation in the rat lower colon. The DCN has a close relationship with colitis-induced pelvic visceral pain. Our results also suggest that NK1R antagonists can provide an anti-nociceptive effect, which might supply experimental evidence for clinical visceral pain therapy.

## Supporting Information

Figure S1 **The total number of visceral pain behaviors after formalin instillation (“For”, ▪), saline instillation (“Sal”, •) and non noxious visceral stimulation (“Nai”, ▴).** **P<0.001 formalin instillation *vs.* saline instillation or naïve group. n = 6 in each group. Values are means ± S.E.M.
(TIF)

Figure S2 **The total number of visceral pain behaviors after saline pretreatment (“S+F”, ▪), L732138 pretreatment (“L+F”, •), L732138 post treatment (“F+L”, ▴) and non treatment (“For”, ▾) under the condition of formalin induced colitis formalin instillation.** **P<0.001 L732138 pretreatment *vs.* saline pretreatment or L732138 post treatment or formalin instillation; ^##^ *P*<0.001 L732138 post treatment *vs.* saline pretreatment or L732138 pretreatment or formalin instillation. n = 6 in each group. Values are means ± S.E.M.
(TIF)

Figure S3 **The co-localization (arrowheads in A”–C”) of NK1R (A–C) and Fos (A’–C’) in dorsal commissural nucleus (DCN, A–A”), lumbosacral spinal dorsal horn (SDH-L, B–B”) and thoracolumbar spinal dorsal horn (SDH-T, C–C”) at 60 min after formalin instillation.** Scale bars, 50 µm. Neurons showing both NK1R-ir and Fos-ir constitute about 20.1%, 20.6% or 21.7% of the total population of Fos-ir neurons in the DCN, SDH-L or SDH-T, respectively, and about 85.4%, 72.2%, and 76.4% of the total population of NK1R-ir neurons in the DCN, SDH-L or SDH-T, respectively (Numbers of neuronal cell bodies in 6 sections through the lumbosacral spinal cord, Table).
(TIF)

File S1 **The experimental procedures of the total number of visceral pain behaviors measurement and co-localized staining of NK1R and Fos.**
(DOC)
