# Supplementary figures and images for: Acute Colitis Induces Neurokinin 1 Receptor Internalization in the Rat Lumbosacral Spinal Cord
Source: PLoS One. 2013 Mar 21;8(3):e59234. doi: 10.1371/journal.pone.0059234 (PMC3605455; doi:10.1371/journal.pone.0059234)

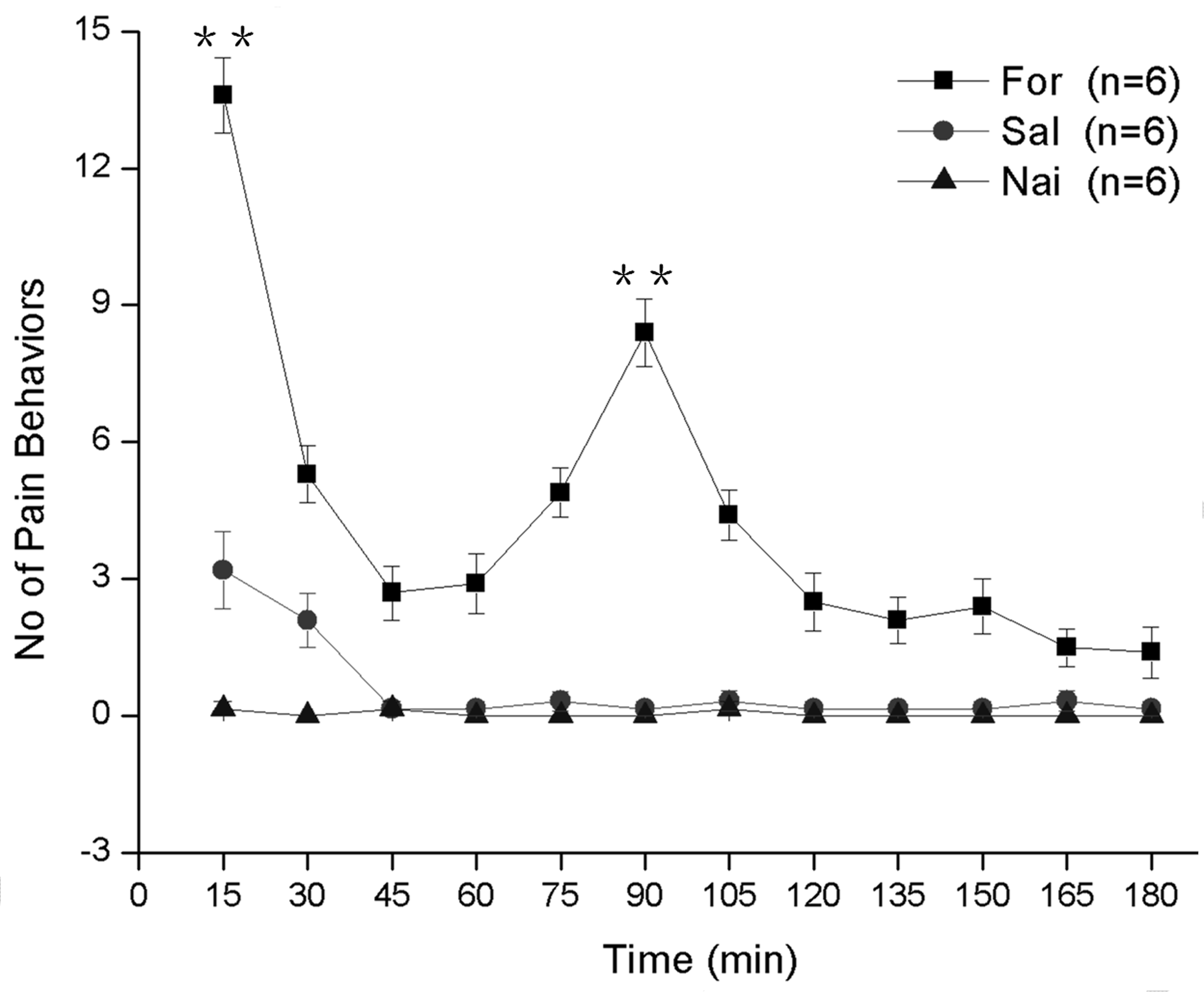

Supplement: Figure S1 — The total number of visceral pain behaviors after formalin instillation (“For”, ▪), saline instillation (“Sal”, •) and non noxious visceral stimulation (“Nai”, ▴). **P<0.001 formalin instillation vs. saline instillation or naïve group. n = 6 in each group. Values are means ± S.E.M. (TIF) [file pone.0059234.s001.tif]

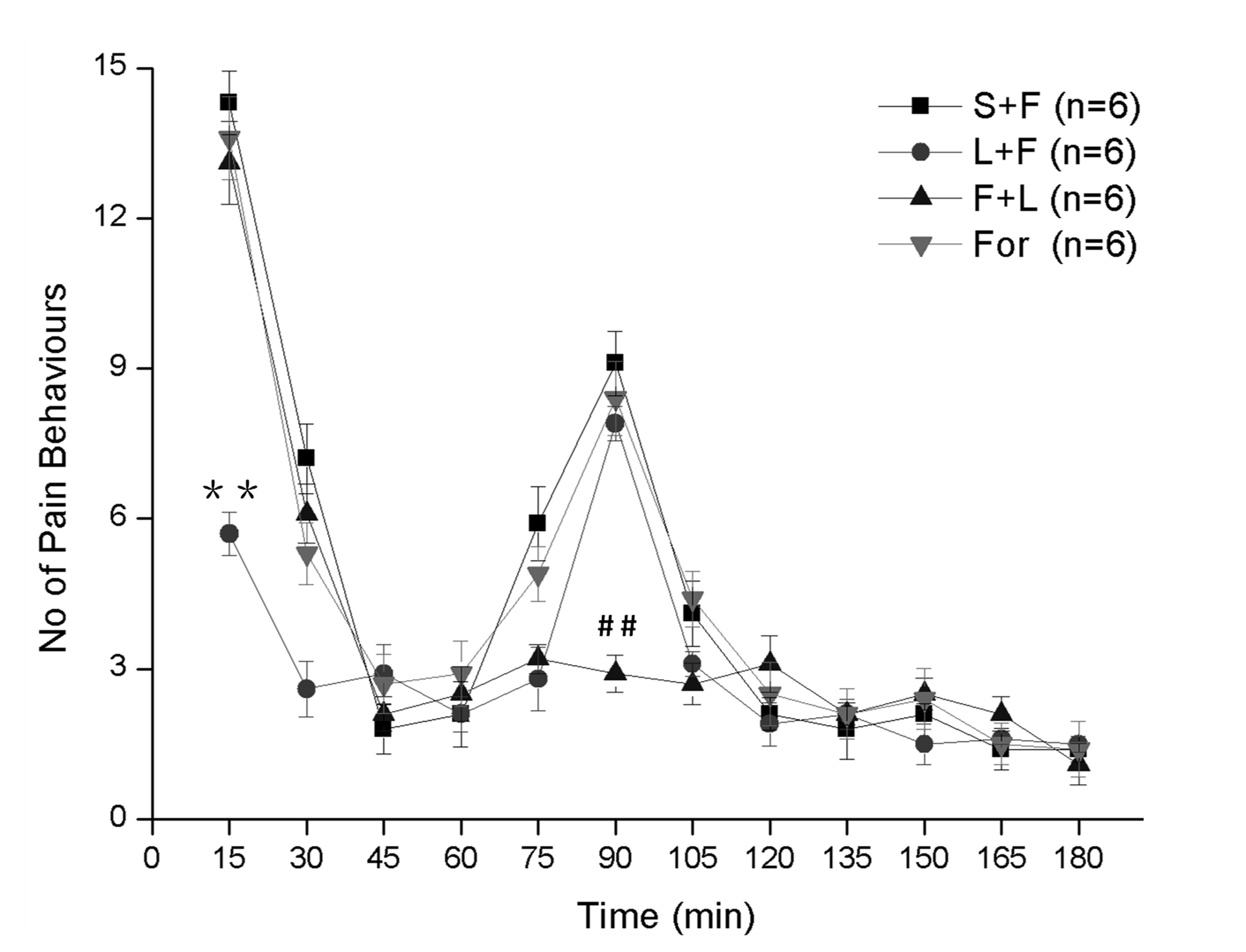

Supplement: Figure S2 — The total number of visceral pain behaviors after saline pretreatment (“S+F”, ▪), L732138 pretreatment (“L+F”, •), L732138 post treatment (“F+L”, ▴) and non treatment (“For”, ▾) under the condition of formalin induced colitis formalin instillation. **P<0.001 L732138 pretreatment vs. saline pretreatment or L732138 post treatment or formalin instillation; ## P<0.001 L732138 post treatment vs. saline pretreatment or L732138 pretreatment or formalin instillation. n = 6 in each group. Values are means ± S.E.M. (TIF) [file pone.0059234.s002.tif]

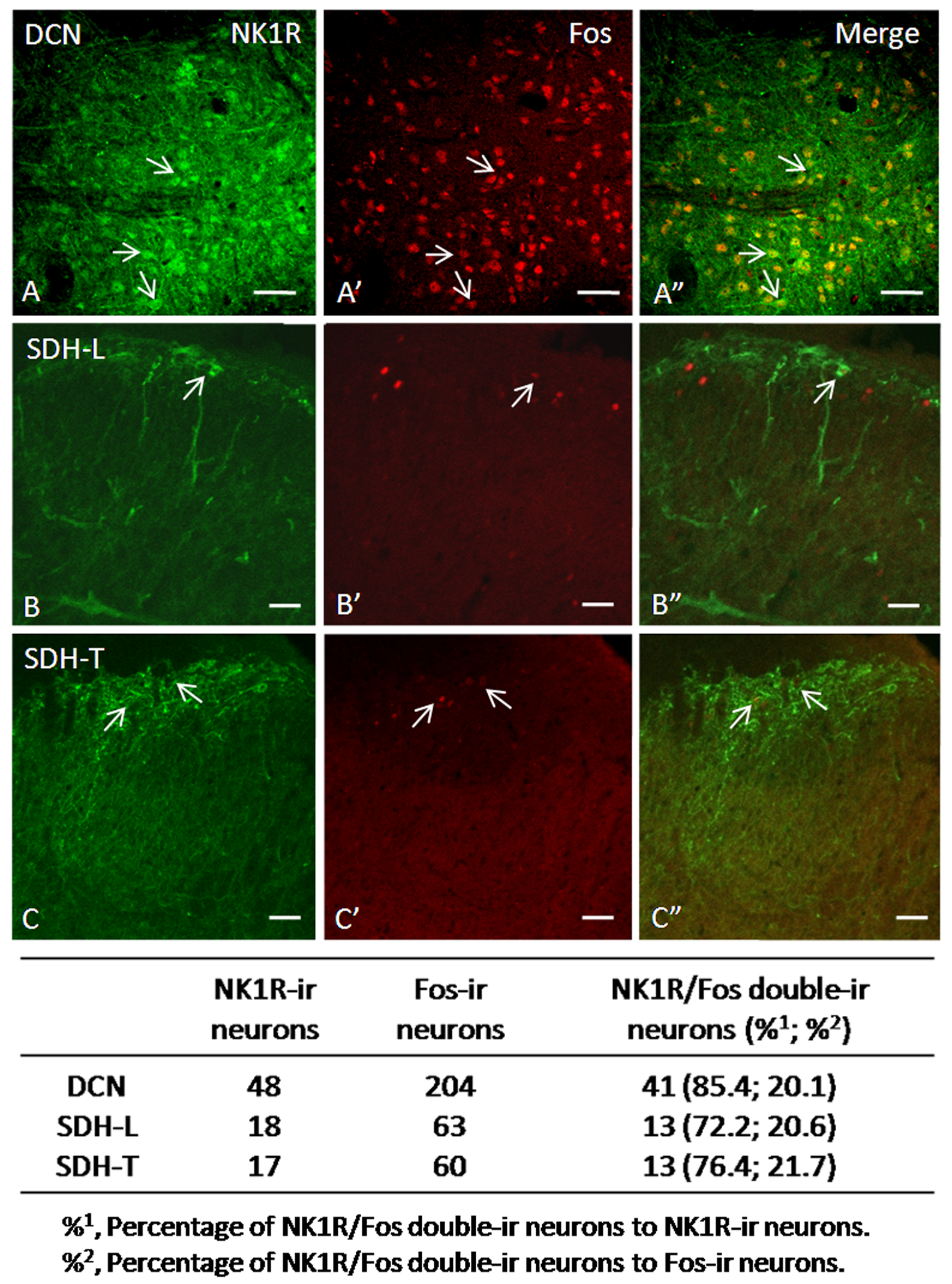

Supplement: Figure S3 — The co-localization (arrowheads in A”–C”) of NK1R (A–C) and Fos (A’–C’) in dorsal commissural nucleus (DCN, A–A”), lumbosacral spinal dorsal horn (SDH-L, B–B”) and thoracolumbar spinal dorsal horn (SDH-T, C–C”) at 60 min after formalin instillation. Scale bars, 50 µm. Neurons showing both NK1R-ir and Fos-ir constitute about 20.1%, 20.6% or 21.7% of the total population of Fos-ir neurons in the DCN, SDH-L or SDH-T, respectively, and about 85.4%, 72.2%, and 76.4% of the total population of NK1R-ir neurons in the DCN, SDH-L or SDH-T, respectively (Numbers of neuronal cell bodies in 6 sections through the lumbosacral spinal cord, Table). (TIF) [file pone.0059234.s003.tif]
