# Supplementary material for: Acute Colitis Induces Neurokinin 1 Receptor Internalization in the Rat Lumbosacral Spinal Cord
Source: PLoS One. 2013 Mar 21;8(3):e59234. doi: 10.1371/journal.pone.0059234 (PMC3605455; doi:10.1371/journal.pone.0059234)
Supplement: File S1 — The experimental procedures of the total number of visceral pain behaviors measurement and co-localized staining of NK1R and Fos. (DOC) [file pone.0059234.s004.doc]

**The total number of visceral pain behaviors**

As we found that the frequency of long time whole body contraction (marked with W2 and W3 in pain score formula:$\text{PS }=\text{ 1L }+\text{2B}+\text{3C}+\text{4W1}+\text{5W2}+\text{6W3}$) in the first phase (30min after formalin instillation) is significantly higher than the second phase (90min after formalin instillation). This explains why the peak of the second phase is small. To confirm the occurrence of second pain phase, we added the total number of visceral pain behaviors. The rats were randomly divided into 6 treatment groups and marked with “For” “Sal” “L+F” “F+L” “S+F” or “Nai”. The “L+F” “F+L” and “S+F” groups received intrathecal catheterization 5 d prior to the formalin instillation. All animals were then placed into observation boxes for 30 min to adapt to the surrounding environment prior to the experiment. The PE tube was connected with a 50 μl volume microinjector. For the “L+F” and “F+L” groups, 10 microliters of vehicle (70 % DMSO; Sal) containing L732138 (100 nmol) were slowly injected, then the tubes were flushed with 5 μl saline. The injection process was finished within 1 min. The “S+F” was given an equal dose of saline as intrathecal vehicle treatment control group. The “For” group was given 100 μl of 5% formalin into the colon, and the “Sal” group was given an equal dose of saline as the negative control. The “L+F” group was pretreated with L732138 (100 nmol/10 μl) 10 min prior to formalin administration, the “S+F” group was pretreated with saline 10 min prior to formalin administration and the “F+L” group was post-treated with L732138 60 min after formalin administration; The “Nai” group was used as a negative control with no drug applied. The rats were then observed for 3 h to record visceral pain-related behaviors.

During this time the total number of visceral pain behaviors was measured. Postures defined as visceral pain-related behaviors were stretching, abdominal retractions and/or abdominal withdrawal reflex.

**Co-localization of NK1R and Fos**

At 60 min after formalin instillation, the rats in formalin instillation group were deeply anesthetized with an overdose of sodium pentobarbital (100 mg/kg, i.p.) and perfused with 100 ml of 0.9% saline, followed by 500 ml of 0.1 M PB containing 4% (w/v) paraformaldehyde. After perfusion, the brains and spinal cords were removed immediately and placed into 0.1 M PB containing 30% (w/v) sucrose overnight at 4°C, and cut into 20 μm-thick serial frontal sections on a freezing microtome (Kryostat 1720; Leitz, Mannheim, Germany). The sections were collected respectively at the levels of lumbosacral and thoracolumbar, divided into 2 series and then washed with 0.01 M phosphate buffer solution (PBS, pH 7.4). The first series of sections were incubated with rabbit anti-NK1R antiserum (1:1000; Chemicon, Temecula, CA) and mouse anti-Fos antiserum (1:500; Sigma, MO, USA) in PBS containing 5% (v/v) normal donkey serum (NDS), 0.3% (v/v) Triton X-100, 0.05% (w/v) NaN_3_ and 0.25% (w/v) carrageenan (PBS-NDS, pH 7.4) overnight at 4°C. After washing with 0.01 M PBS, the sections were incubated with Alexa 594-conjugated donkey anti-mouse IgG (1:500; Sigma) and Alexa 488-conjugated donkey anti-rabbit IgG (1:500; Sigma) secondary antibodies in PBS containing 5% normal goat serum and 0.2% Triton X-100 for 2 h. The sections were washed with 0.01 M PBS 3 times for 10 min each between each step. Finally, the slides were mounted onto gelatin-coated glass slides, air-dried and cover-slipped with a mixture of 50% (v/v) glycerin and 2.5% (w/v) triethylene diamine (anti-fading agent) in 0.01 M PBS. Images were obtained using a confocal laser scanning microscope (FV1000; Olympus, Tokyo, Japan).

The second series of sections were incubated with normal rabbit and mouse serum instead of the primary antibodies followed by the same staining protocol to serve as negative controls. The intensity of the staining was quantified using densitometry by experimenters who were blind to the treatment conditions.
